# Detection of the Lactate Threshold in Runners: What is the Ideal Speed to Start an Incremental Test?

**DOI:** 10.1515/hukin-2015-0022

**Published:** 2015-04-07

**Authors:** José Luiz Dantas, Christian Doria

**Affiliations:** 1 Functional Evaluation Laboratory, Department of Neurosciences, Imaging and Clinical Sciences, ‘Gabriele d’Annunzio’ University of Chieti–Pescara, Italy.

**Keywords:** initial speed, blood lactate concentration, aerobic threshold, endurance running

## Abstract

Incremental tests on a treadmill are used to evaluate endurance athletes; however, no criterion exists to determine the intensity at which to start the test, potentially causing the loss of the first lactate threshold. This study aimed to determine the ideal speed for runners to start incremental treadmill tests. The study consisted of 94 runners who self-reported the average speed from their last competitive race (10–42.195 km) and performed an incremental test on a treadmill. The speeds used during the first three test stages were normalised in percentages of average competition speed and blood lactate concentration was analysed at the end of each stage. The relationship between speed in each stage and blood lactate concentration was analysed. In the first stage, at an intensity corresponding to 70% of the reported average race speed, only one volunteer had blood lactate concentration equal to 2 mmol·L^−1^, and in the third stage (90% of the average race speed) the majority of the volunteers had blood lactate concentration ≥2 mmol·L^−1^. Our results demonstrated that 70% of the average speed from the subject’s last competitive race – from 10 to 42.195 km – was the best option for obtaining blood lactate concentration <2 mmol·L^−1^ in the first stage, however, 80% of the average speed in marathons may be a possibility. Evaluators can use 70% of the average speed in competitive races as a strategy to ensure that the aerobic threshold intensity is not achieved during the first stage of incremental treadmill tests.

## Introduction

Incremental tests using analyses of blood lactate concentration (BLC) are widely used to evaluate endurance athletes (Castagna et al., 2013; Faude et al., 2009). They provide individual physiological thresholds that serve to prescribe an optimum training intensity workload (Faude et al., 2009; Guellich et al., 2009; Seiler and Kjerland, 2006) and track physiological adaptations caused by long-term exercise (Castagna et al., 2013; Faude et al., 2009; McMillan et al., 2005). A fixed value (e.g., 2.0 or 2.5 mmol·L^−1^) or a change in relation to the rest concentration (i.e., Δ from 0.2 to 1.0 mmol·L^−1^, ≈2 mmol·L^−1^) are considered as the first lactate threshold (LT1) which represents the aerobic threshold (Faude et al., 2009; Lucia et al., 1999). The LT1 is the first important index obtained from an incremental test for three reasons: (1) it reflects submaximal aerobic capacity (Yoshida et al., 1987) and any offset to the right indicates an improvement in this capacity (Faude et al., 2009); (2) speed at the LT1 is among one of the best predictors of long distance running performance (Bassett and Howley, 2000; Yoshida et al., 1987); (3) endurance athletes should spend a large amount of their training time at intensity equal to or lower than LT1 intensity (Esteve-Lanao et al., 2005; Esteve-Lanao et al., 2007). Consequently, athletes and coaches need to know the intensity corresponding to the LT1 in order to control and monitor training sessions.

Incremental tests have already established a general standard protocol and resolved some important points, such as: step durations (Kuipers et al., 2003; Machado et al., 2013; Weltman et al., 1990); increments (Berthon and Fellmann, 2002; Kuipers et al., 2003); pauses (Berthon and Fellmann, 2002); and submaximal (McMillan et al., 2005) and maximal termination criteria (Berthon and Fellmann, 2002). However, the ideal intensity at which to start the test remains unclear, particularly when the LT1 needs to be determined. In order to adequately establish the LT1, initial intensity of a test needs to be lower than the workload corresponding to BLC of 2 mmol·L^−1^. In reality, the level of intensity at which to start incremental tests using BLC analysis is not a recent problem and some solutions have been proposed, e.g., a percentage of maximal power output/speed (Beneke et al., 2011) or a percentage of the maximal oxygen consumption/heart rate (Berthon and Fellmann, 2002; McMillan et al., 2005). However, as it is unfeasible in a real environment to perform an additional test to obtain the individual maximal parameters, they are frequently unknown prior to the incremental test. Consequently, researchers have admitted to either choosing the initial speed subjectively, using their experience to estimate the aerobic capacity of a volunteer (Almarwaey et al., 2004; Held and Marti, 1999) or fixing an initial speed for all volunteers arbitrarily (Castagna et al., 2013; Kuipers et al., 2003; Machado et al., 2013).

The aforementioned discussion highlights the importance of the initial speed in incremental protocols. However, evidence supporting this choice is non-existent, making it difficult to justify and establish an objective criterion. On one hand, as previously reported, misjudged intensity at the onset of the test may induce higher BLC than the LT1, causing this threshold to be missed (Davis, Caiozzo et al., 1983). On the other hand, very low speed at the onset of the test necessitates the use of many lactate samples, making the test expensive. Determining a criterion that can be obtained from competition data could be an alternative solution (e.g., average speed). Average speed in competitions is independent of individual maximal parameters, easy to calculate and frequently known by amateur runners. Thus, this study aimed to determine the ideal speed at which to start incremental tests on a treadmill for runners, using average competition speed.

## Material and Methods

### Participants

Retrospective data (2011 – 2012) were used in this study. The data came from incremental tests, which were performed by amateur male runners in our laboratory (n = 94; age = 41.1 (6.9) years; body mass = 71.5 (7.1) kg; body height = 1.76 (0.62) m and the body mass index = 23.0 (1.9) kg/m^2^). All volunteers were healthy without a history of cardiovascular diseases and were physically active performing regular endurance training (≥3 times per week, training experience from 1 to 5 years) during the preparation phase of their training schedule. All participants in the study had taken part in at least one competitive race (10–42.195 km) and one incremental test in our laboratory in the year prior to the test; data from the subjects without a competitive race and a laboratory test were excluded. To ensure an adequate nutritional profile and rest on the day of the test, the volunteers were asked to avoid strenuous exercise and alcohol beverages (minimum 24 h), and beverages and foods containing caffeine (minimum 3 h). Moreover, they were asked to ingest a light meal at least 2 hours prior to the test and sleep for a minimum of 8 h during the night preceding the assessment session. All procedures in this study were conducted in accordance with the Declaration of Helsinki and all participants signed a written informed consent form approved by the Institutional Review Board of the ‘Gabriele d’Annunzio’ University of Chieti–Pescara.

### Procedures

The volunteers performed a submaximal incremental test (stages = 4 min; increments = 1.5 km·h^−1^; end of the test = BLC ≥4 mmol·L^−1^; intervals between stages for BLC collection = 30 s) on a treadmill (RunRace, Technogym, Gambettolla (FC), Italy). The speed of the first stage for each volunteer was chosen according to their estimated cardiorespiratory fitness. This estimate was determined by two experienced evaluators based on each volunteer’s background training, conforming to the usual methods used in the laboratory and practical settings. Each volunteer reported their average speed(s) and the distance(s) performed in their last competition(s) (range 10 to 42.195 km), which is a common procedure during the pre-test interview in our laboratory. The evaluators were advised to maintain a conservative stance in relation to the choice of initial speed, i.e., if they had a doubt between two speeds, they were advised to choose the lower. This procedure was implemented to avoid BLC ≥2 mmol·L^−1^ in the first stage causing the loss of LT1 data. The warm-up consisted of a 6 min run at a speed corresponding to 1.5 km·h^−1^ below the chosen speed for the first stage. A 30-s interval was allowed between stages for blood sampling. Capillary blood samples (5 μl) were taken from the earlobe of the volunteers, BLC being analysed immediately using a portable analyser (Lactate Pro LT-1710, Arkray, Kyoto, Japan). The device was calibrated before each test according to the manufacturer’s instructions.

### Analysis

Descriptive statistics were used to describe the data from the first, second and third stages of the incremental test. Normality was verified using the *Shapiro Wilk* test. Data are presented as mean and standard deviation (SD), a confidence interval (95% CI) and minimal and maximal values for every stage. For each volunteer, the speeds used during the test stages were normalised in percentages of the average speed performed in the last competition (%AVS_Race_). When a volunteer had performed more than one competition over different distances, the lowest %AVS_Race_ was used for the analyses. The data were categorised for analyses. The mean value of the speed in each stage was rounded to the nearest 10 percent, creating the cut offs to categorise this variable. After categorisation, the cut offs were 70, 80 and 90%AVS_Race_ for the first, second and third stages, respectively. BLC of 2 mmol·L^−1^ was used as a cut off for this variable. Speed and BLC data from each volunteer in each stage were categorised in relation to %AVS_Race_ (< and ≥ the speed cut off for the stage) and BLC (<2 mmol·L^−1^ and ≥2 mmol·L^−1^). In addition, BLC of 1 mmol·L^−1^ (<1 mmol·L^−1^ and ≥1 mmol·L^−1^) was used as a cut off for the first stage to verify whether the speed corresponding to 70%AVS_Race_ had underestimated the ideal speed to start the test. The Chi-square test for contingency tables (2 × 2) was used to verify relationships between BLC and speed in each stage. The Fisher’s exact test was used when the expected frequency was lower than 5 cases in the Chi-square test. All data were analysed using Microsoft Excel 2010 software (Microsoft Corporation, WA, USA) and SPSS 17.0 statistical software (SPSS, Inc.®, Chicago, IL, USA). The significance level was set at *p* < 0.05.

## Results

Among the 94 volunteers, the frequency of participation in marathons (42.195 km), half-marathons (21.097 km) and 10 km races was 73, 88 and 48, respectively. The average speeds in the races were 12.5 (1.6), 13.8 (1.4) and 14.7 (1.7) km·h^−1^ for the marathon, half-marathon and 10 km races, respectively. The %AVS_Race_ for each race distance is shown in Table 1. The values of BLC were 1.1 (0.2), 1.4 (0.4) and 2.2 (0.7) mmol·L^−1^ for the 1^st^, 2^nd^ and 3^rd^ stages, respectively.

**Figure 1:**
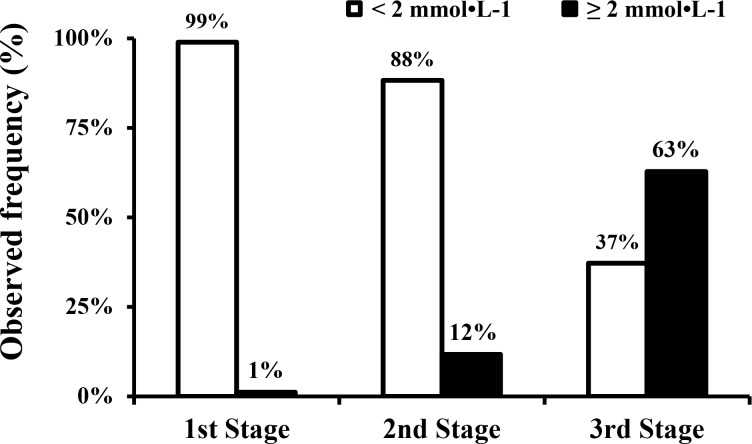
Observed frequency (%) of blood lactate concentration presented after each stage using the cut-off point of 2 mmol·L^−1^.

In the first stage, only one volunteer presented BLC exactly equal to 2 mmol·L^−1^, and in the third stage, the majority of the volunteers presented BLC ≥2 mmol·L^−1^ (Figure 1). BLC was higher than 1 mmol·L^−1^ in 79 of the 94 volunteers during the first stage (<70%AVS_Race_ = 42; ≥70%AVS_Race_ = 37). The majority of volunteers who had a speed <70%AVS_Race_ in the first stage (37 of 48) presented BLC ≥1 mmol·L^−1^. The volunteer who had BLC equal to 2 mmol·L^−1^ belonged to this <70%AVS_Race_ group (1^st^ stage= 85.9% and 68.5%AVS_Race_ performed in marathon and half marathon races, respectively).

No relationship was found in the first stage between speed (<70 or ≥70%AVS_Race_) and BLC (<2 and ≥2 mmol·L^−1^) (Fixer’s Exact test; *p =* 0.553), or for BLC of 1 mmol·L^−1^ cut-off (<1 and ≥1 mmol·L^−1^) (Fixer’s Exact test; *p =* 0.607). The second stage demonstrated a significant relationship (*X^2^*= 4.100; *p* = 0.043), showing that speed ≥80%AVS_Race_ tends to produce BLC ≥2 mmol·L^−1^ more often than speed <80%AVS_Race_. In the same way, the third stage demonstrated a significant relationship (*X^2^*= 8.960; *p* = 0.003), showing that speed ≥90%AVS_Race_ of competitions causes a high frequency of BLC ≥2 mmol·L^−1^. The %AVS_Race_ values in each stage in relation to BLC responses are shown in Table 2.

## Discussion

This study aimed to determine the ideal speed at which to start incremental treadmill tests for runners. Our results demonstrated that 70%AVS_Race_ in the last competition – from 10 to 42.195 km – was the best option for obtaining BLC below 2 mmol·L^−1^ in the first stage.

This study provides an efficient strategy to ensure BLC below the LT1 in the first stage of treadmill incremental tests. Review studies have standardized the procedures and parameters for incremental tests (Beneke et al., 2011; Bentley et al., 2007; Berthon and Fellmann, 2002; Faude et al., 2009; Myers and Bellin, 2000). However, they have not presented a criterion for choosing the initial speed for treadmill tests. Beneke et al. (2011) arbitrarily suggested that incremental tests should start at moderate exercise intensity, but without detailing any objective parameter. If chosen arbitrarily, a given initial speed can cause loss of the LT1 data. Data from previous studies have shown that the initial speed chosen for incremental tests is sometimes very close to the speed corresponding to the LT1 (Castagna et al., 2013; Manzi et al., 2009). For instance, in the study of Castagna et al. (2013), the speed range at BLC of 2 mmol·L^−1^ was from 10.9 to 12 km·h^−1^, and the treadmill test started at a speed of 9 km·h^−1^. In the same way, in the study of Manzi et al. (2009), the mean speed at BLC of 2 mmol·L^−1^ was 11.1 (0.7) km·h^−1^ whilst the initial speed of the test was 10 km·h^−1^. These studies did not mention lost data due to the fact that the protocol started at a speed very close to the LT1 intensity, but considering a 95% confidence interval, it is likely that some athletes may have reached BLC ≥2 mmol·L^−1^ in the first stage, as already mentioned in a previous study (Davis, Caiozzo et al., 1983). Thus, our study provides an objective criterion for choosing the intensity to start an incremental treadmill test, avoiding an overestimation of initial speed.

An initial speed close to 70%AVSRace did not underestimate the ideal speed to start incremental tests when the reference was the speed in half marathons or 10 km races, but it could underestimate the ideal speed if the reference was the speed in marathons. However, the volunteer who had BLC exactly equal to 2 mmol·L^−1^ presented an initial speed lower than 70%AVSRace in relation to his highest race speed. Therefore, if the evaluators had chosen to start the test at an intensity corresponding to 70%AVSRace before the experiment, they would have ensured BLC <2 mmol·L^−1^ for the majority of volunteers (99%). Furthermore, our results showed that a speed lower than 70%AVSRace can produce BLC ≥1 mmol·L^−1^, evidencing that this intensity did not underestimate the ideal initial speed for the incremental test. Actually, the majority of volunteers showed BLC >1 mmol·L^−1^ during the first stage, even though many of them performed at a speed <70%AVSRace. It is worth mentioning that, in Table 1, lower limits of the confidence interval and a minimal value obtained in relation to the marathon speed were lower than 80%AVSRace in the first stage. In short, the probability of reaching BLC <2 mmol·L^−1^ using a speed of 70%AVSRace was high: i.e., 1 evaluated subject in every 100 evaluations could show BLC higher than the desired concentration in the first stage. Thus, our results suggest that 70%AVSRace is a conservative option to ensure an intensity lower than the LT1.

A speed corresponding to 80%AVSRace reduced the probability of reaching BLC <2 mmol·L^−1^ to 88%: i.e., 3 in every 25 would show BLC higher than the desired concentration for the first stage. This is an unacceptable rate in relation to the previous 99%. Furthermore, the association between speed ≥80%AVSRace and BLC ≥2 mmol·L^−1^ highlights the risk of overestimation, although only 11 of the 94 volunteers presented BLC ≥2 mmol·L^−1^ during the second stage. Consequently, 80%AVSRace could also be an option to start incremental treadmill tests. However, this intensity can induce BLC above the LT1 more often than 70%AVSRace, mainly if the adopted concept of the LT1 is an alteration in relation to basal concentrations. The lower limits of the confidence interval and minimal speed values in Table 2 showed that some volunteers reached BLC ≥2 mmol·L^−1^ at an intensity below 80%AVSRace in the second stage. Thus, 70%AVSRace is still a better option than 80%AVSRace to ensure BLC under LT1 intensity in the first stage.

A speed of 90%AVSRace is definitively not an option for starting incremental tests aimed at detecting the LT1 because the majority of volunteers showed BLC ≥2 mmol·L^−1^ at this intensity (63% of tested subjects). The synthesis of our results suggest adopting an initial speed for incremental tests of 70%AVSRace as the ideal and 80%AVSRace as an option when the reference is the average speed performed in marathons. However, our results discourage adopting a speed corresponding to 90%AVSRace as a parameter to start incremental tests on a treadmill. The LT1 and performance in competition data obtained from previous studies corroborate our data (Castagna et al., 2013; Manzi et al., 2009; Roecker et al., 1998). Athletes’ data obtained from the study of Roecker et al. (1998) showed that the speed at the LT1 corresponded to 71.8, 74.6 and 83.4%AVSRace of the speed in 10 km, half marathon and marathon races, respectively. Additionally, data from the study of Manzi et al. (2009) showed that initial speed for the test and speed at BLC ≥2 mmol·L^−1^ in amateur runners corresponded to 81.5 and 90.4%AVSRace of speed in a marathon, respectively. These studies corroborate our results demonstrating that the secure option for starting incremental tests is 70%AVSRace calculated from 10 km or half marathon races and 80%AVSRace calculated from marathon races.

Our criterion to start incremental treadmill tests could also be utilized for professional runners, since their thresholds correspond to an intensity ≥70% of their maximal speed in incremental tests (Rabadan et al., 2011). Consequently, these threshold intensities represent speeds higher than 70%AVSRace. Furthermore, we suggest that our criterion could also be utilized when using ventilatory analysis. Since the first ventilatory and the first lactate thresholds are physiologically related (Davis, Bassett et al., 1983), the same strategy of starting at 70%AVSRace is probably a secure option to keep the first stage intensity lower than the first ventilatory threshold. Consequently, evaluators and coaches can use 70%AVSRace as a strategy to ensure that an intensity corresponding to the LT1 is not achieved during the first stage of incremental treadmill tests using blood lactate concentration or ventilatory analyses in runners.

In conclusion, the speed corresponding to 70%AVSRace is the ideal and conservative option to ensure a first stage intensity lower than the LT1 during incremental tests on a treadmill. A speed corresponding to 80%AVSRace could be an option when the reference is the average speed in marathon races.

## Figures and Tables

**Table 1:** Percentage of average speed performed in each stage according to the distance of competitive races

Stage	RACE km	Mean (SD)	95% CI	Minimal	Maximal
		%AVS_Race_	%AVS_Race_	%AVS_Race_	%AVS_Race_
1^st^					
	10 km	68 (4)	60 to 75	60	79
	21.5 km	71 (4)	63 to 79	63	81
	42.195 km	79 (5)	69 to 89	69	90
	The lowest %AVS_Race_	69 (4)	61 to 77	60	79
2^nd^					
	10 km	78 (5)	69 to 88	68	90
	21.5 km	82 (5)	73 to 92	73	95
	42.195 km	92 (6)	79 to 104	79	104
	The lowest %AVS_Race_	80 (5)	71 to 90	68	91
3^rd^					
	10 km	88 (5)	78 to 99	77	98
	21.5 km	93 (5)	82 to 104	82	105
	42.195 km	104 (8)	89 to 119	87	118
	The lowest %AVS_Race_	91 (6)	80 to 102	77	103

***%AVS_Race_*** = *percentage of average speed in the last competitive race;* ***SD*** = *standard deviation;* ***95% CI*** = *confidence interval;* ***The lowest %AVS_Race_*** = *the average using the lowest %AVS_Race_ from each volunteer*

**Table 2:** Percentage of average speed performed in each stage according to blood lactate concentration

Stage	BLC	Participants	Mean (SD)	95% CI	Minimal	Maximal
	(mmol·L^−1^)	n	%AVS_Race_	%AVS_Race_	%AVS_Race_	%AVS_Race_
1^st^						
	≤ 1	79	69 (4)	61 to 77	60	79
	>1 and < 2	14	71 (4)	64 to 79	64	79
	≥ 2	1	69 ----	---- to ----	----	----
2^nd^						
	≤ 1	14	78 (6)	67 to 89	68	88
	>1 and < 2	69	80 (5)	71 to 89	71	91
	≥ 2	11	83 (4)	75 to 91	75	89
3^rd^						
	≤ 1	1	82 ----	---- to ----	----	----
	>1 and < 2	34	89 (6)	77 to 101	77	103
	≥ 2	59	92 (5)	83 to 102	81	102

***BLC*** = *blood lactate concentration;* ***%AVS_Race_*** = *percentage of average speed in the last competitive race;*

***SD*** = *standard deviation;* ***95% CI*** = *confidence interval*
